# Clinical and biometrological efficacy of a hyaluronic acid-based mesotherapy product: a randomised controlled study

**DOI:** 10.1007/s00403-013-1360-7

**Published:** 2013-05-29

**Authors:** Martine Baspeyras, Céline Rouvrais, Laetitia Liégard, Alexandre Delalleau, Sandrine Letellier, Irène Bacle, Laetitia Courrech, Pascale Murat, Valérie Mengeaud, Anne-Marie Schmitt

**Affiliations:** 1 Dermatologist, Bordeaux, France; 2 Pierre Fabre Dermo-Cosmétique, European Centre for Skin Research, Hôtel-Dieu Saint-Jacques, 2 rue Viguerie, BP 3071, 31025 Toulouse Cedex 3, France

**Keywords:** Mesotherapy, Hyaluronic acid, Dermis thickness, Micro-injection

## Abstract

Data demonstrating the efficacy of hyaluronic acid (HA)-based mesotherapy for skin rejuvenation are scarce. The aim of the study is to assess the efficacy of non-reticulated HA-based mesotherapy on skin elasticity and complexion radiance. 55 women with cutaneous ageing signs included in the Full Analysis Set (FAS) population blindly received intradermal micro-injections (50 × 0.02 mL) of non-cross-linked HA filler with mannitol (Glytone 1, HA concentration: 14 mg/g) in one cheek and saline physiological solution in the other according to hemifacial randomisation in 3 monthly sessions. Elasticity (E1 and E2 stiffness parameters) and dermis thickness were measured by cutometry and 20 MHz echography, before (D0) treatment and 1 (1M) and 3 months (3M) after the last injection. A trained panel blindly scored skin complexion radiance from standardised and calibrated photographs, using 100 mm analogue scales. In the FAS population, only HA filler significantly decreased E1 at 1M (−10.9 %, *p* = 0.026) and 3M (−10.5 %, *p* = 0.035) compared with D0; its effect versus the control tended to be more persistent, with a difference between treatments at 3M close to significance (*p* = 0.063). E2 also decreased at 1M (−8.2 %, *p* = 0.027 in the per protocol population, *n* = 53) and 3M after HA-treatment only. Dermis thickness significantly increased after HA-treatment at 1M (+3.4 %, *p* = 0.028) and 3M (+4 %, *p* = 0.008), and after control-treatment at 1M only (+2.5 %, *p* = 0.015). The HA filler significantly improved complexion radiance at 3M compared with the control (*p* = 0.012) and for 51 % of subjects, their skin status. Non-reticulated HA-based mesotherapy significantly and sustainably improves skin elasticity and complexion radiance.

## Introduction

With age and UV exposure, skin undergoes morphologic and mechanical changes that manifest as wrinkling, sagging, loss of elasticity and dryness [9]. In particular, decreased synthesis of collagen and elastin and their increased degradation, reduced proliferative capacity of fibroblasts and perturbations in the organisation of elastic fibre network lead to alterations in the mechanical properties of the skin with reduced resilience and elasticity [10, 19]. Advances in the knowledge of the biochemical mechanisms associated with ageing have led to the development of different approaches to reduce and repair its untoward effects [15], particularly by using minimally invasive procedures.

Originally developed to treat vascular and lymphatic disorders, mesotherapy has recently been used for skin rejuvenation. The method consists in multiple and micro-dosed injections of bioactive products into the skin to increase its hydration and reconstruct an optimal physiological environment for the fibroblasts. It is aimed in particular at enhancing cell activity and synthesis of collagen, elastin and hyaluronic acid (HA) [11]. The most common formulation of mesotherapy for facial skin rejuvenation includes repetitive injections of a multivitamin solution in the superficial dermis [4]. However, among the products available for skin rejuvenation by mesotherapy, HA plays an important role in the hydration of the extracellular space due to its ability to attract water molecules and it is thought to create the physiological conditions in the extracellular matrix for proliferation, migration and organisation of dermal cells [8]. Clinical experience of skin rejuvenation by HA-based mesotherapy suggests this technique is safe inasmuch as it is performed by a trained physician, who follows safe-injection practices with appropriate aseptic techniques to prevent the risk of infection related to inadequate safety measures. Furthermore, several studies suggest it can improve skin hydration, firmness and viscoelastic properties [12, 13, 16]. However, published results from clinical studies demonstrating the efficacy of this approach in improving viscoelastic mechanical properties of the skin are scarce.

The main objective of this study was therefore to assess the effect of intradermal microinjections of a non-cross-linked HA-based mesotherapy product with mannitol (Glytone^®^ professional 1), on mechanical properties of facial skin compared with a control product in subjects displaying mild to moderate cutaneous ageing signs. With this aim, we performed measurements by cutometry of mechanical parameters at the dermis level, which is assumed to be the main structure involved in viscoelastic mechanical properties of the skin [17, 18], and we measured the thickness of the dermis as a secondary endpoint. Other objectives of this study were to compare the effects of HA filler and control on skin complexion, their efficacy self-evaluated by the subjects, and their tolerance.

## Subjects and methods

This clinical, biometrological, single-blind, randomised study was carried out at the Centre de Recherche sur la Peau Pierre Fabre (CRP), Toulouse (France), according to the ethical principles of the declaration of Helsinki and the guidelines for Good Clinical Practices (CPMP/ICH/135/95). The protocol was approved by the Committee for the Protection of Persons South-West and Overseas III and the French Agency for the Safety of Health Products (AFSSAPS). Each volunteer signed a written informed consent.

### Subject selection

Female volunteers (30–65 years), of phototypes I–III according to Fitzpatrick classification, with mild to moderate cutaneous ageing signs (mild to visible dehydration, mild to marked sagging/slackening, sallow and/or olive-greenish complexion, no wrinkles to wrinkles at rest and fine lines on the surface) on the face were included. Non-menopausal women had to be under effective contraception since at least 2 months before inclusion and had to have negative pregnancy test results at inclusion and each mesotherapy session. Menopause diagnosis was to be confirmed in menopausal women. Subjects presenting the following criteria were not included: pregnant or breast-feeding women, any cutaneous pathology of infectious, inflammatory, viral and vascular type affecting the face, auto-immune and granulomatous pathologies, diabetes, Osler’s endocarditis, wound healing disorders, allergy history to HA or any ingredient of the test product or to any other product used in the study (Anesderm^®^ and Septeal^®^, Pierre Fabre Dermatologie, Boulogne-Billancourt, France; Glytone^®^ Suncare and Glytone^®^ Post-Op, Pierre Fabre Dermatologie Esthetique, Boulogne-Billancourt, France). Other criteria for non-inclusion were the regular use of hormone or systemic (retinoid–based products, immunosuppressants, steroid anti-inflammatory drugs) or local (highly active topical corticoids) treatments that could influence the study results within the 3 months before inclusion, any peeling within the 2 years before inclusion, and any mesolift, lifting, botulinum toxin treatment, HA or other filler injections, previous facial surgery, remodelling or ablative laser procedures within the year before inclusion.

### Treatments

The study product was a HA injectable solution (Glytone^®^ 1 professional, Merz Pharmaceutical GmbH, Frankfurt, Germany) consisting of 14 mg/mL non-animal non-reticulated HA in phosphate sodium thus providing a viscosity suitable for microinjections and increasing the half-life of the molecule. The HA injectable solution contains glycerol, a strong moisturising agent which also plays a role in skin elasticity and potentiates the action of HA, and mannitol which in particular is known for limiting the degradation of HA by free radicals through its antioxidant action. The product was provided in a pack of two pre-filled sterile 1-mL glass syringes supplied with two 30G ½ needles. Physiological saline solution (Aguettant^®^) was used as a control product.

### Study design

The study consisted of three injection sessions at monthly intervals and three assessment visits, the first one 14 days before the first injection (D0), the second 1 month (1M) after the last injection session and the third 3 months (3M) after the last injection session. The same trained investigator administered HA and control products to all subjects. One hour before each injection session, the subject applied a topical local anaesthetic cream (Anesderm^®^) on the face. After having removed the cream, the whole face was then thoroughly cleaned with a disinfecting lotion (Septeal^®^). During each treatment session, the subject randomly received about 50 × 0.02 mL of study product in one side of the face and the same amount of control product in the other side, to allow intra-individual comparison and thus overcome the problem of inter-individual variations in skin status. Both products were injected manually into the lower part of the cheek, at the level of the dermis/epidermis junction or/and the superficial dermis using the gold standard serial puncture technique [21], as described by Iorizzo et al. [11]. A pattern was applied to the skin to precisely mark injections points, thus allowing reproducibility of serial injections at each time-point. The intervention was single blinded, as the subject was not informed of the injection side of the study product. The difference in viscosity between the study and control products did not allow the investigator performing the injections to be unaware of treatment assignments. Then, the investigator massaged the injection sites to ensure the placement of the product, and reminded the subject of precautions to follow after injection (no makeup during the following 12 h, no exposure to extreme temperatures). The subjects had then to apply a soothing cream (Post-op Glytone^®^) twice a day during the five following days. They were also instructed to avoid sun exposure during the study and were provided a sunscreen (Glytone^®^ Suncare) to apply in case of exposure.

### Skin elasticity and dermis thickness measurements

Skin elasticity was measured using a Cutometer^®^ MPA580 (Courage & Khazaka, Cologne, Germany). The device generated negative pressure of up to 100 mbar at a rate of 20 mbar/s sucking up the skin into a probe of 6 mm diameter aperture, which was put in contact with the skin perpendicularly to the surface. When the pressure was withdrawn at the same rate, the skin returned to its normal shape. The movement of the skin in and out the probe is illustrated in a pressure–deflexion curve, which allows the determination of skin stiffness and viscosity parameters. These parameters were determined using a nonlinear skin behaviour model specifically developed by the Pierre Fabre Skin Research Centre [7] and requiring measurements of dermis thickness. Dermis thickness was measured by echography using a high-frequency (20 MHz) ultrasound scanner (Dermcup, ATYS Medical, Soucieu en Jarrest, France) [14]. E1 and E2 parameters define the stiffness of elastin and collagen fibres, respectively, and the more they decrease the more skin is compliant. Equivalent strain parameter (*ε*_eq_) corresponds to the strain necessary for collagen fibres extension.

Biometrological measurements were performed at two pre-specified 2 cm areas on both cheeks in controlled conditions at 20 ± 4 °C during the three evaluation visits following a 15 min rest of the subject.

### Skin complexion assessment

Skin complexion was evaluated by a panel of 16 experts trained to do the quantitative descriptive analysis. Using this methodology, they had to describe complexion radiance and uniformity with the help of calibrated and standardised photographs of the hemiface, which were cropped to only display the treated area of the cheek. In addition, photographs were rendered anonymous and randomised to allow blind scoring by the panel. The complexion evaluation was based on the identification and the selection of descriptors for establishing a sensory profile [1, 2]. Fifteen training sessions were necessary to validate panel performance, i.e. the reproducibility, discrimination and reliability of the panellists in descriptive tests. The sensory profile was composed of eight items, one for the global assessment of complexion radiance and seven for the multidimensional analysis of complexion: four items detailed complexion radiance (uniformity, hydration, yellow and pink aspects of the complexion) and three items described general skin complexion (firmness, luminosity/brightness, and quantity of wrinkles). For each hemiface of each subject, randomised cropped photographs corresponding to the three evaluation time-points were presented to the panellists, who blindly scored the eight items using 100 mm visual analogue scales (e.g. from 0 = not radiant at all to 100 = very radiant).

### Evaluation criteria

#### Primary efficacy criterion

The change in cutaneous mechanical properties induced by the product was evaluated by assessing skin elasticity parameters at the level of the product- and control-treated hemifaces, at D0 and 1 month after the last session of mesotherapy (1M).

### Secondary efficacy criteria

Change in skin elasticity at the level of the control and HA-treated hemifaces was also assessed 3 months after the last session of mesotherapy (3M) to evaluate the persistence of the product effect.

Change in dermis thickness was also evaluated by echography at the level of the control and HA-treated areas on the cheeks at D0, 1 and 3M.

The efficacy of the product was also evaluated on skin complexion by scoring its radiance and uniformity at the level of the control and HA-treated hemifaces from standardised and calibrated photographs at D0, 1 and 3M.

Finally, the subjects self-assessed the global efficacy of the product after each mesotherapy session and at 1 and 3M by using a 5-point scale (aggravation, no improvement, slight, moderate and important global improvement).

### Safety assessment

All adverse events (AE) occurring at inclusion and throughout the study were reported from the first evaluation visit (D0). Local tolerance was evaluated after all injection visits and at 3M using a 4-point scale from 1 = very good tolerance (no functional or objective symptom) to 4 = poor tolerance (functional and/or objective symptoms leading to treatment discontinuation).

### Statistical analyses

Statistical analysis was performed by using SAS^®^ software. Quantitative variables were described by number of subjects, mean and standard deviations, median and minimum and maximum, qualitative variables, by number of subjects and percentage for both groups of treatment. The main analysis was carried out on the Full Analysis Set (FAS) population; secondary analyses were carried out on per protocol (PP) population.

The main efficacy criterion was assessed by analysis of covariance (ANCOVA) with the product, site and sequence factors as fixed effects, the subject factor as random effect and the baseline as the covariate. When the product effect was significant, the comparison between control and product effect was performed at each time-point. The same analyses were used for the secondary criteria except for self-assessment of product efficacy, which was analysed at each time point by the Wilcoxon’s signed rank test. Significance level was 5 % for the whole study.

## Results

### Subjects’ flow and demographic characteristics

A total of 60 women were included in the study. Five subjects were excluded from efficacy analysis, three because they did not receive any injection and two because they were not evaluated for the main efficacy criterion. The FAS population therefore included 55 subjects (97 %) aged 34–65 years (mean age 52.4 years). Two subjects were excluded from the PP population due to a major protocol deviation (hormonal therapy stopped 1 week before inclusion, inversion of randomisation), which consequently consisted of 53 subjects aged 34–65 years (mean age 52.1 years). Results were reported for the FAS population only, except when different results were obtained in the PP population.

### Primary efficacy criterion

#### Effect of control and HA filler on skin elasticity at 1M

Skin elasticity parameters measured by cutometry at the level of the control and HA-treated cheek areas in the FAS population are shown in Table 1. At D0, skin elasticity parameters were not significantly different between control and HA-treated cheek areas.

**Table 1:** Skin elasticity parameters before (D0) and 1 (1M) and 3 months (3M) after the end of mesotherapy sessions in control- and HA filler HA product-treated hemifaces in the FAS population

Cutometry parameter (mean ± SD)	D0	1M	% of change 1M vs D0	3M	% of change 3M vs D0
E1 (MPa)					
HA product	0.149 ± 0.063	0.133 ± 0.054	−10.9*	0.132 ± 0.053	−10.5*
Control	0.134 ± 0.051	0.131 ± 0.052	−2.3	0.138 ± 0.057	+4.5
*p* value^‡^	NS	NS	–	0.0634 (SL)	–
E2 (MPa)					
HA product	0.558 ± 0.175	0.518 ± 0.161	−6.8^†^	0.529 ± 0.174	−4.3
Control	0.551 ± 0.190	0.530 ± 0.161	−4.1	0.544 ± 0.182	−0.7
*p* value^‡^	NS	NS	–	NS	–
*ε*_eq_					
HA product	0.056 ± 0.017	0.063 ± 0.015	+13.5***	0.062 ± 0.014	+10.5**
Control	0.058 ± 0.013	0.063 ± 0.015	+8.8**	0.060 ± 0.016	+3.6
*p* value^‡^	NS	NS	–	NS	–
Dissipation					
HA product	0.751 ± 0.037	0.745 ± 0.054	−0.94	0.755 ± 0.047	+0.2
Control	0.752 ± 0.038	0.753 ± 0.044	+0.15	0.754 ± 0.049	+0.3
*p* value^‡^	NS	NS	–	NS	–

*NS* non-significant, *SL* significance limit

E1 and E2 parameters define elastin and collagen fibres stiffness, respectively, and the more they decrease the more skin is compliant. Equivalent strain parameter (*ε*_eq_) corresponds to the strain necessary for collagen fibres extension

* Comparison versus D0, *p* < 0.05

** Comparison versus D0, *p* < 0.01

*** Comparison versus D0, *p* < 0.001

^†^Comparison versus D0, slightly significant difference, *p* = 0.065

^‡^Comparison between control and HA product treatments

Between D0 and 1M, E1 parameter significantly decreased by 10.9 % in the HA-treated hemifaces (*p* = 0.026), whereas it did not significantly change in the control hemifaces (−2.3 %, *p* = 0.260) (Table 1). Compared with D0, HA-based treatment also induced a decrease in E2 parameter by −6.8 % at 1M, although it was not significant (*p* = 0.065) (Table 1). However, when the product effect was analysed in the PP population, E2 parameter change between D0 and 1M reached statistical significance (−8.2 %, *p* = 0.027). By contrast, the control did not induce any significant change between D0 and 1M, whatever the population analysed (FAS or PP). Nevertheless, the comparison between HA and control treatments failed to show any significant difference in E1 and E2 parameters (Table 1, FAS population). With regard to *ε*_eq_ parameter, both HA-based and control treatments induced a significant increase (+13.5 % *p* = 0.0002 and +8.8 %, *p* = 0.0014, respectively) at 1M versus D0 in the FAS population, without significant difference between both treatment effects (Table 1).

### Secondary efficacy criteria

#### Effect of control and HA filler on skin elasticity at 3M

Results of HA and control treatments on skin elasticity 3 months after the last mesotherapy session in the FAS population are shown in Table 1. HA effect on skin elasticity persisted 3 months after the end of mesotherapy sessions with a decrease of E1 and E2 parameters at 3M compared with D0 by −10.5 % (*p* = 0.035) and −4.3 % (*p* = 0.297), respectively; whereas, the control did not show any significant effect on E1 and E2 parameters. Furthermore, compared with the control, HA effect tended to be more persistent, since the difference between the two treatments at 3M was close to significance (*p* = 0.063). The remanence of HA effect was also significant on collagen fibre entanglement, with a 10.5 % increase of *ε*_eq_ parameter at 3M compared with D0 (*p* = 0.002), whereas the effect of the control was not significant anymore. However, the difference in *ε*_eq_ parameter between HA and control treatments was not significant. The time-effect of control and HA treatment on dissipation was not significant.

#### Effect of control and HA filler on dermis thickness

Results of control and HA filler effects on dermis thickness before and 1 and 3M after the end of mesotherapy in the FAS population are shown in Table 2. Compared with D0, dermis thickness significantly increased 1 and 3M after the last injection of HA (+3.4 %, *p* = 0.028 and +4 %, *p* = 0.008, respectively). The control also induced a significant increase of dermis thickness at 1M compared with D0 (+2.5 %, *p* = 0.015), but this effect did not persist at 3M (1.1 %, *p* = 0.179). No significant difference was observed between control and HA effects.

**Table 2:** Dermis thickness measured by echography before (D0), and 1 month (1M) and 3 months (3M) after the last injection session of control and HA filler in the FAS population

Dermis thickness, mm (mean ± SD)	D0	1M	% of change D0–1M	3M	% of change D0–3M
HA filler	1.674 ± 0.214	1.731 ± 0.192	+3.4*	1.741 ± 0.195	+4**
Control	1.716 ± 0.225	1.759 ± 0.221	+2.5*	1.735 ± 0.205	+1.1
*p* value^†^	0.122	–	0.842	–	0.290

Results are expressed as mean ± SD of 300 ultrasound images

* Comparison versus D0, *p* ≤ 0.05

** Comparison versus D0, *p* ≤ 0.01

^†^Comparison between control and HA treatments using ANCOVA analysis on the changes at 1 and 3M

#### Effect of control and HA filler on complexion

Before the first session of mesotherapy (D0), the scores for the eight items of skin complexion were not significantly different between control and HA-treated hemifaces.

HA filler had a significant effect on complexion radiance at 3M compared with D0 (*p* = 0.023), and it was significantly more efficient than the control, with an improvement of complexion radiance by +6.2 % from D0 in HA-treated hemifaces versus impairment by −2.3 % from D0 in the control-treated hemifaces (*p* = 0.012). Among the seven items describing skin complexion, yellow aspect, and skin hydration improved 1M after the end of mesotherapy in HA and control hemifaces (Table 3). Pink aspect also transiently improved at 1M in the control hemiface (*p* = 0.041). However, the effects of the HA filler persisted at 3M, whereas the control effect was not maintained (Table 3). HA filler effect on yellow aspect and hydration tended to be significant at 1M (*p* = 0.057 and *p* = 0.088) and 3M (*p* = 0.055 and *p* = 0.076), whereas control effect was significant at 1M only (*p* = 0.025 and *p* = 0.022). However, no significant difference between control and HA effects was observed on these parameters.

**Table 3:** Skin complexion parameters evaluated by an expert panel before (D0) and 1 (1M) and 3 months (3M) after the end of mesotherapy sessions in control and HA filler-treated hemifaces

Score (mean ± SD)	D0	1M	% of change D0–1M	3M	% of change D0–3M
Complexion radiance					
HA product	42.9 ± 9.4	44.6 ± 9.7	+3.9	45.6 ± 11.4	+6.2*
Control	43.2 ± 8.5	44.0 ± 9.2	+2.1	42.2 ± 9.4	−2.3
*p* value^‡^	0.710	–	0.621	–	0.012
Yellow aspect					
HA product	15.6 ± 8.8	13.8 ± 7.8	−11.3^†^	13.8 ± 9.0	−11.4^†^
Control	14.9 ± 7.8	13.1 ± 7.1	−12.1*	14.8 ± 8.7	−0.4
*p* value^‡^	0.201	–	0.782	–	0.175
Pink aspect					
HA product	33.6 ± 11.2	35.6 ± 11.8	+5.9	35.7 ± 12.1	+6.2
Control	34.5 ± 13.3	36.8 ± 12.9	+6.8*	35.0 ± 11.1	+1.5
*p* value	0.243	–	0.637	–	0.403
Skin firmness					
HA product	59.4 ± 14.1	58.6 ± 14.9	−1.3	59.8 ± 14.3	+0.7
Control	61.1 ± 13.5	60.8 ± 13.8	−0.6	61.3 ± 14.1	+0.2
*p* value^‡^	0.051	–	0.398	–	0.898
Hydration					
HA product	48.7 ± 8.2	50.1 ± 9.3	+2.9^†^	50.2 ± 9.5	+3.0^†^
Control	47.9 ± 8.2	50.2 ± 8.3	+4.6*	48.1 ± 9.2	+0.4
*p* value ^‡^	0.172	–	0.604	–	0.142
Luminosity					
HA product	43.3 ± 9.2	44.7 ± 10.6	+3.3	44.9 ± 11.1	+3.8
Control	44.2 ± 9.7	45.1 ± 9.3	+2.0	43.1 ± 9.5	−2.4
*p* value^‡^	0.184	–	0.985	–	0.102
Wrinkle quantity					
HA product	26.0 ± 15.6	25.7 ± 15.6	−1.2	25.2 ± 15.1	−2.9
Control	24.4 ± 14.1	23.6 ± 14.3	−3.3	23.1 ± 14.7	−5.7^†^
*p* value^‡^	0.086	–	0.396	–	0.330
Evenness					
HA product	43.4 ± 12.7	44.5 ± 13.4	+2.6	44.4 ± 14.7	+2.3
Control	42.5 ± 10.7	42.8 ± 11.7	+0.8	41.6 ± 11.9	−2.1
*p* value^‡^	0.297	–	0.501	–	0.157

Each score was calculated by using a 100 mm visual analogue scale and was expressed as the mean ± SD of the scores obtained by the 16 assessors. Improvement in pink aspect, skin firmness, hydration, luminosity and evenness was characterised by an increase of the score, that of yellow aspect and wrinkle quantity by a decrease of the score

* Comparison versus D0, *p* ≤ 0.05

^†^Comparison versus D0, *p* ≤ 0.1

^‡^Comparison between HA and control treatments using ANCOVA analysis on the changes at 1 and 3M

#### Self-assessment of product efficacy

**Fig. 1:**
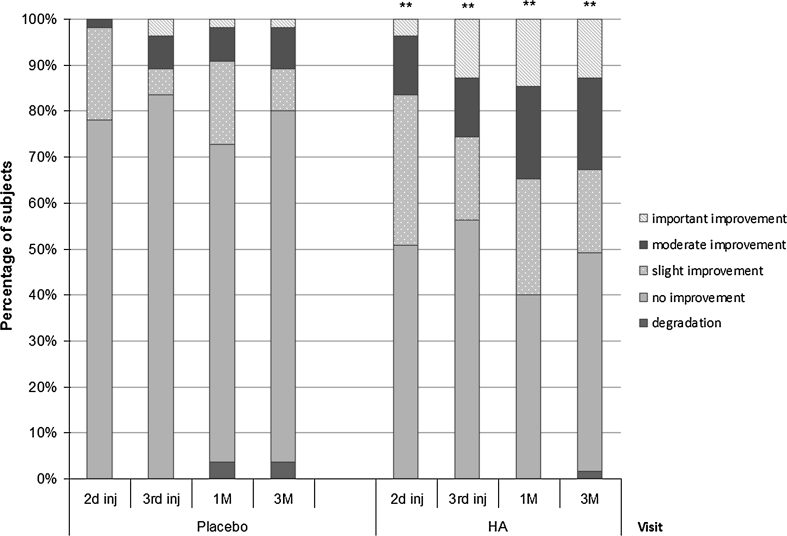
Perception by the subjects of HA-based mesotherapy efficacy compared with the placebo 1 month after first, second and third injection visits (2nd inj, 3rd inj and 1M) and 3 months after the third injection (3M). The subject assessed the global product efficacy on each hemiface by using a 5-point scale (worsening, stable, slight improvement, moderate improvement, high improvement). Comparison between HA and placebo effects was performed using the Wilcoxon’s test, **p ≤ 0.01

At all time points of evaluation, the percentage of subjects having perceived an improvement of their skin status in the HA-treated hemiface compared with the control-treated one was significantly higher (*p* ≤ 0.01) (Fig. 1). Ever since the first month after the first mesotherapy session (2nd injection visit), 16.5 % of the subjects perceived a moderate to important improvement at the level of the HA-treated hemiface, whereas they were <2 % to find a moderate improvement in the control-treated hemiface. This improvement of the HA-treated side increased with treatment duration and persisted 3 months after the end of the third mesotherapy session: a moderate to important improvement was observed by 34.6 and 32.8 % of subjects in the HA-treated hemiface, at 1 and 3M, respectively, versus 9 and 10.9 % of subjects in the control-treated hemiface.

#### Safety and tolerance assessment

Among the 57 subjects included in the tolerance study population, 50 (87.7 %) experienced one or several adverse effects after injection. Adverse events were generally of mild or moderate intensity and expected (haematoma, oedema, papule, erythema or other transient inflammatory reaction): 46.8 % occurred in the HA-treated hemiface and 25.7 % in the control. The most commonly expected adverse event was haematoma, both in the HA and control-treated hemifaces, with a global incidence of 35.7 and 20.5 %, respectively. Four subjects experienced severe haematoma after injection in the HA-treated hemiface, and four others reported a non-expected severe adverse effect (pregnancy, otitis, shoulder tendonitis aggravation, functional ankle impairment), but none was related to the study product. All expected adverse events disappeared within a mean time of 5.9 days.

Local tolerance to both products was good to excellent in 85–100 % of cases throughout the study.

## Discussion

This controlled single-blind study using hemifacial treatment randomisation demonstrated the efficacy of a non-cross-linked HA-based mesotherapy product with mannitol (Glytone^®^ professional 1) in improving dermis mechanical behaviour and complexion radiance of facial skin with a 3-month remanence. In contrast to the control, intradermal microinjections of HA filler induced a significant decrease of E1 and E2 parameters, indicating a decrease of elastin and collagen fibre stiffness and suggesting that this product may significantly improve skin compliance. This effect persisted 3 months after the last mesotherapy injections for E1 parameter and tended to be significant compared to the control. Furthermore, HA injections significantly and sustainably increased *ε*_eq_ parameters in contrast to the control, the effect of which was smaller and did not persist with time. This suggests that non-reticulated HA-based mesotherapy may sustainably regain suppleness to the skin and increase entanglement of its collagen fibres thus restoring the mechanical behaviour of a young skin.

The significant effect of HA-based mesotherapy on skin elastic properties was coupled in our study with a sustained increase in dermis thickness 1 and 3M after the last mesotherapy session. With the control, a significant effect was observed at 1M only. This transient dermis thickening with both treatments at 1M may be explained by the fact that the mechanical stimulation of microinjections induced dermis micro-inflammation with vasodilatation persisting 1 month after the last injection session. However, as dermis thickening remained significant 3M after the HA treatment only, we may hypothesise that by contrast with the control, HA injections secondarily induced the synthesis of dermis components such as elastin and collagen which may contribute to dermis thickness increase and be responsible for the remanence of this effect. Collagen and elastin fibre synthesis activation and their potential renewal may also explain the effect of HA on skin elastic parameters. The newly formed fibres may be more compliant and their greater number and entanglement may reinforce the collagen and elastin network, which is embedded in a proteoglycan and glycosaminoglycan gel to form the dermis. The results of a placebo-controlled study supports this hypothesis, since it demonstrated that injections of stabilised HA into the forearm skin significantly increased the synthesis of type 1 collagen in addition to profibrotic growth factors [20]. Another study, which observed an increase in the echogenicity of the subepidermal low-echogenic band by ultrasound analysis after HA-based mesotherapy every week for 4 weeks on the dorsum of the hands, also suggested these changes may be related to an increased density of dermal collagen fibres by fibroblast activation resulting from treatment [13].

Another part of our study was the blinded assessment of skin complexion radiance on standardised and calibrated photographs. For this study, we developed a reliable and reproducible method to objectively describe skin complexion and in particular complexion radiance. To prevent the panellists from being visually influenced by the skin aspect of the non-treated areas, skin complexion was evaluated using randomised and cropped photographs corresponding to the treated area of the cheeks and not to the whole hemiface. With this method, we demonstrated for the first time a positive effect of mesotherapy on skin complexion radiance: HA microinjections induced a significant and sustainable improvement in complexion radiance compared with the control. Other skin complexion parameters such as yellow aspect and hydration also tended to improve after HA injections.

Finally, HA-based mesotherapy was considered globally efficient by the subjects, as the percentage of the subjects having perceived an improvement after each injection session into the HA-treated hemiface was significantly higher compared with the control-treated hemiface. Particularly, compared with the control, the percentage of subjects having perceived slight to important improvement 1 and 3 months after the third injection of HA was more than twice higher (60 vs 27.3 % and 51 vs 20 %, respectively). HA injections were well tolerated, with a good to excellent local tolerance in >85 % of cases.

Altogether, our results confirm those of previous studies showing that non-reticulated HA-based mesotherapy can improve skin hydration, firmness and viscoelastic properties. Although our study failed to show higher efficacy of HA filler compared with placebo in improving parameters of dermis mechanical behaviour and skin complexion, its significant effect on skin complexion radiance and the fact that E1 parameter was borderline significantly improved at 3M suggest that statistical significance might have been achieved in a larger study population.

Only one non-comparative pilot study evaluating the effect of three HA gel microinjection sessions on skin elasticity and dermal thickness of 19 women has been published [12, 16]. Using the cutometry and echography methods, the authors showed a significant increase in all skin elasticity parameters (gross and net elasticity, skin extensibility, relaxation, fatigability and capacitance) in both cheeks 1 and 3 months after the last injection [12, 16], but they failed to show changes in skin thickness [16].

Another study evaluating the effect of microinjections of a vitamin/HA solution by histology and electron microscopy of skin biopsies also failed to show changes in epidermal and dermal thickness [4]. This absence of clinical and histological modification may be due to a too small amount of non-reticulated HA injected into the skin by each micropuncture. The injection material was composed of a 9:1 suspension of a multivitamin solution in a non-conjugated HA gel, but unfortunately its concentration was not indicated, precluding any comparison.

With regard to skin stiffness, Reuther et al. [16] suggested an increase of this parameter, whereas we have shown a significant decrease. Although microinjections were performed at the level of the mid-dermis using a mesotherapy technique, the study product was a non-animal stabilised HA (NASHA) gel and cutometry measurements were performed with a probe with a 2 mm aperture and by applying a 450 mbar negative pressure, i.e. experimental conditions which allow epidermis and stratum corneum evaluation. From a mechanical point of view, dermis is the structure assumed to exert the greatest influence on elastic mechanical properties of the skin [5, 22], a point which was recently confirmed by comparisons between finite element models and elastographic measurements [6]. We therefore considered that cutometry measurements at the dermis level were more adapted to the objectives of our study. This is why, according to Agache et al. [3], we used a 6-mm aperture probe to specifically measure dermal mechanical parameters and we coupled these measurements with dermis thickness assessments by echography to allow calculation of intrinsic cutometry parameters [7]. In addition, our study was controlled against physiological saline solution and the products were injected randomly in the two hemifaces to avoid the drawback of inter-individual variations.

In conclusion, this study objectively demonstrated the efficacy and the tolerance of a non-cross-linked HA filler in sustainably improving skin elastic parameters and complexion radiance. In particular, we showed that intradermally microinjected HA might be of value to improve suppleness of ageing skin, inasmuch as injections are performed by a trained physician with appropriate aseptic measures. It might be worthy of a further study to assess the role of HA in decreasing stiffness parameters and to determine if its action is mainly mechanical or mediated by the activation of dermis components synthesis by fibroblasts.
